# Testing algal-based *p*CO_2_ proxies at a modern CO_2_ seep (Vulcano, Italy)

**DOI:** 10.1038/s41598-020-67483-8

**Published:** 2020-06-29

**Authors:** Caitlyn R. Witkowski, Marcel T. J. van der Meer, Nadine T. Smit, Jaap S. Sinninghe Damsté, Stefan Schouten

**Affiliations:** 1 Department of Marine Microbiology and Biogeochemistry, NIOZ Royal Netherlands Institute for Sea Research, and Utrecht University, PO Box 59, 1790AB Den Burg (Texel), The Netherlands; 2 Department of Geosciences, Faculty of Earth Sciences, Utrecht University, PO Box 80.021, Utrecht, The Netherlands; 3 Present Address: University of Bristol, School of Earth Sciences, Wills Memorial Building, Queens Road, Bristol, BS8 1RJ UK

**Keywords:** Biogeochemistry, Climate sciences, Environmental sciences

## Abstract

Understanding long-term trends in atmospheric concentrations of carbon dioxide (*p*CO_2_) has become increasingly relevant as modern concentrations surpass recent historic trends. One method for estimating past *p*CO_2_, the stable carbon isotopic fractionation associated with photosynthesis (Ɛ_p_) has shown promise over the past several decades, in particular using species-specific biomarker lipids such as alkenones. Recently, the Ɛ_p_ of more general biomarker lipids, organic compounds derived from a multitude of species, have been applied to generate longer-spanning, more ubiquitous records than those of alkenones but the sensitivity of this proxy to changes in *p*CO_2_ has not been constrained in modern settings. Here, we test Ɛ_p_ using a variety of general biomarkers along a transect taken from a naturally occurring marine CO_2_ seep in Levante Bay of the Aeolian island of Vulcano in Italy. The studied general biomarkers, loliolide, cholesterol, and phytol, all show increasing depletion in ^13^C over the transect from the control site towards the seep, suggesting that CO_2_ exerts a strong control on isotopic fractionation in natural phytoplankton communities. The strongest shift in fractionation was seen in phytol, and *p*CO_2_ estimates derived from phytol confirm the utility of this biomarker as a proxy for *p*CO_2_ reconstruction.

## Introduction

The concentration of atmospheric carbon dioxide (*p*CO_2_, expressed in partial pressure µatm), as directly measured from air trapped in ice cores, has had a major influence on climate over the past 800 thousand years (ka)^1^. During this period, *p*CO_2_ and temperature oscillated together between stable bounds every 100 ka^2^. In the past two centuries, the rise of *p*CO_2_ has broken those bounds from the pre-industrial values, previously only ranging between ca. 180 to 280 µatm, to the 410 µatm of today^3^. This rapid rise in *p*CO_2_ causes concern that climate, particularly temperature, will accordingly change. In order to better understand how changes may occur, reconstructing longer trends in *p*CO_2_ over the geologic record could offer context for evaluating the direction and magnitude of climate change.

Many proxies have been developed for reconstructing past *p*CO_2_ and applied with mixed success over the past several decades^4^. One method for studying past *p*CO_2_ makes use of the stable carbon isotopic fractionation due to CO_2_-fixation (Ɛ_p_), where biomass of photoautotrophs becomes increasingly depleted in ^13^C as *p*CO_2_ increases due to kinetic discrimination by the CO_2_-fixing enzyme Rubisco^5–7^. Ɛ_p_ can be derived from the δ^13^C of photoautotrophic biomass, recorded in sedimentary organic matter, and the δ^13^C of inorganic CO_2_ derived from the carbonate in the shells of planktonic foraminifera^8^.

Although *p*CO_2_ has been shown to be one of the dominant physiological control on the δ^13^C of photoautotrophic biomass^9^, studies on Ɛ_p_ in algae have shown that other factors may influence this value, primarily growth rate^10^ and cell size and shape^11^, as well as minor influences such as light, and temperature^12–15^. These additional influencing factors on Ɛ_p_ are considered in *p*CO_2_ reconstructions via the catchall term *b*^16^, described in the equation^17^ as:

1 $$ {\text{CO}}_{{{2}[{\text{aq}}]}} = b/ \, (\upepsilon_{{\text{f}}} - \upepsilon_{{\text{p}}} ) $$

where Ɛ_f_ is the maximum isotopic fractionation due to CO_2_-fixation via the enzyme Rubisco, which has shown a sum range from 25 to 28‰^17–19^. It should be noted that the very few in vivo Rubisco fractionation studies have much lower values^20,21^, which Wilkes and Pearson^22^ suggest there may be due to multiple stages of fractionation instead of the singular Rubisco fractionation step. Several other studies have expanded on Eq. (1) for specific consideration, particularly in calculating *b*, e.g. instantaneous cell growth rate accounting for differences in photoperiod^23,24^ and CO_2_ fixation rate^25^.

Using the knowledge obtain from culture studies^26,27^, the measurement of Ɛ_p_ in algal biomarkers preserved in the geologic record can be used to reconstruct past *p*CO_2_. These biomarkers are almost exclusively alkenones, long-chain unsaturated methyl and ethyl *n*-ketones produced by haptophytes^8,28,29^. Although this proxy has generated a large number of *p*CO_2_ records^30–32^, there are several limitations, such as the exceptionally low Ɛ_f_ recorded for the alkenone-producer *Emiliania huxleyi* of 11‰^20^, a potential insensitivity of this proxy at low CO_2_ levels^24,33^, and difficulties in constraining the *b* factor over time^34^. One other limitation is the fact that alkenones first commonly appeared in the geologic record ca. 45 million years (Ma) ago^35^, prohibiting *p*CO_2_ reconstructions prior to this time.

As an alternative, the isotopic fractionation of general phytoplankton biomarkers, compounds that are produced by a multitude of species, may offer some solutions to the limitations of the alkenone *p*CO_2_ proxy such as more spatial ubiquity and temporal longevity. This general biomarker approach has been poorly explored; however, though there are some examples of this being applied to phytane, a diagenetic product of omnipresent chlorophyll-a, for periods extending beyond the alkenone record, i.e. in the Cretaceous^36–38^ and in a Phanerozoic compilation^39^. However, this general biomarker approach has not been extensively tested in laboratory cultures or present-day environments.

For modern studies of the general biomarker approach, naturally-occurring phytoplankton communities are necessary to mimic the widespread contributors to general phytoplankton biomarkers, as opposed to the typical single-species approach of laboratory cultures. Mesocosm experiments may offer more natural environmental conditions and communities, though none have been conducted on general phytoplankton biomarkers for *p*CO_2_ reconstructions. Alkenones and particulate organic carbon (POC) have been explored in one mesocosm experiment using natural communities, i.e. under three *p*CO_2_ conditions in a contained area for ca. 21 days^40^. These authors suggested the minor changes they observed in δ^13^C values for alkenones and POC indicate that fractionation is not primarily controlled by CO_2_ concentrations but instead by algal growth rate and carbon-uptake mechanisms. However, these experiments are inherently difficult to set-up, reproduce, and control.

Here we expand this new approach to testing *p*CO_2_ response in natural phytoplankton communities, by analyzing the response of isotopic fractionation in general phytoplankton biomarkers across a CO_2_ gradient at a naturally occurring CO_2_ seep. CO_2_ seeps, which consistently bubble CO_2_ into the surrounding environment and thus have very high CO_2_ concentrations near the seep, have hardly been explored for biological studies due to the assumed high sulfide concentrations, toxic to many organisms, typically associated with volcanic degassing^41^. However, Hall-Spencer et al.^42^ used these extremely high *p*CO_2_ environments for ocean acidification experiments, which lead to studies at other seep sites, i.e. Italy^43^, Papua-New-Guinea^44^, New Zealand^45^, and Japan^46^. The new approach was initially tested with a 3-point transect (high, mid, and control *p*CO_2_) of a marine CO_2_ seep site on Shikine Island, Japan, covering a range of CO_2_ concentrations that offer an analogue for past oceans^39^. However, this specific site proved to have confounding factors where the imprint of CO_2_ on Ɛ_p_ measured in general biomarkers of surface sediment was masked by extreme weather events (i.e. typhoons) that caused sediment transport.

Here, we more thoroughly explore this new approach at a different marine CO_2_ seep system approximately 30 m into Levante Bay at Vulcano Island, Italy, a location with much more stable weather conditions than Japan. We collected surface sediments in a high-resolution 16-point transect from high CO_2_ towards ambient CO_2_ values. Here, we analyzed the Ɛ_p_ of several general phytoplankton biomarkers, compounds that have been virtually unstudied in modern phytoplankton communities, deposited in surface sediments and tested their response to the CO_2_ gradient at sixteen sites throughout the bay.

## Results

**Figure 1:**
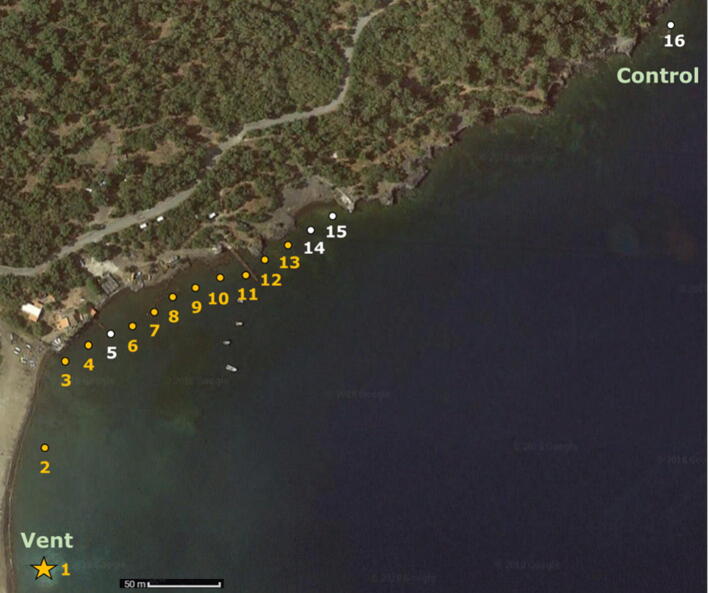
Map of sites in Levante Bay. Sampling sites along the transect from the CO_2_ seep (star, Site 1) to the ambient control (Site 16) on Vulcano Island, Italy (Google Maps). White symbols indicate the additional sampling sites in May 2017.

For this study, we collected surface sediments in May and October from close to the seep site (ca. 3 m distance) to a control site unaffected by the seep^47^ at a constant depth of ca. 1.5 m at the time of sampling (Fig. 1). The δ^13^C of DIC measured in seawater collected in May from the bay does not show notable change over the gradient of CO_2_ (Table S1), which confirms that lack of change noted in the literature^48^. For this reason, we averaged the δ^13^C of DIC measured in our study with that of Cornwall et al.^49^ across all sites (0.7‰ ± 0.4‰ s.d.) and assumed this to be representative for the bay region.

**Figure 2:**
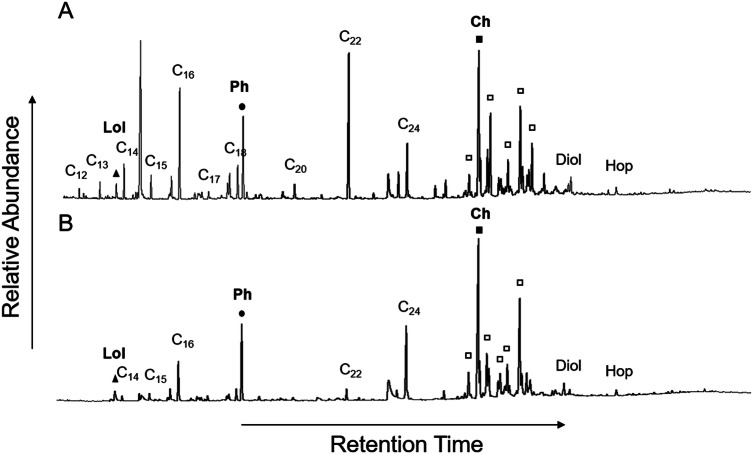
Chromatogram of silylated polar fraction of extract of surface sediments with (**A**) control site with ambient CO_2_ concentrations and (**B**) Site 5 near CO_2_ vent. Major compounds are loliolide (Lol, closed triangle), phytol (Ph, closed circle), cholesterol (Ch, closed square), as well as fatty alcohols (chain-lengths shown), C_30_ alkane-1,15-diol (Diol), C_32_-17β(H),21β-hopanol (Hop), and sterols (squares).

Analysis of the polar fractions of the lipid extracts obtained from the surface sediments showed the same biomarker lipids in similar distributions throughout the transect from the CO_2_ seep to the control sites (e.g. biomarker distributions at Site 5, near the CO_2_ seep, and the control site, Site 16, are shown in Fig. 2). These biomarker lipids include: loliolide, phytol, even carbon numbered C_10_-C_16_ fatty alcohols, C_30_ alkane-1,15-diol, C_32_-17β(H),21β-hopanol, and sterols, such as cholesta-5,22E-dien-3β-ol, cholesterol, 23-methylcholesta-5,22dienol, campesterol, stigmasterol, and β-sitosterol (Fig. 2). Consistently, the most abundant among these compounds were loliolide, cholesterol, and phytol, as were also observed at the CO_2_ seep site in Japan^50^.

**Figure 3:**
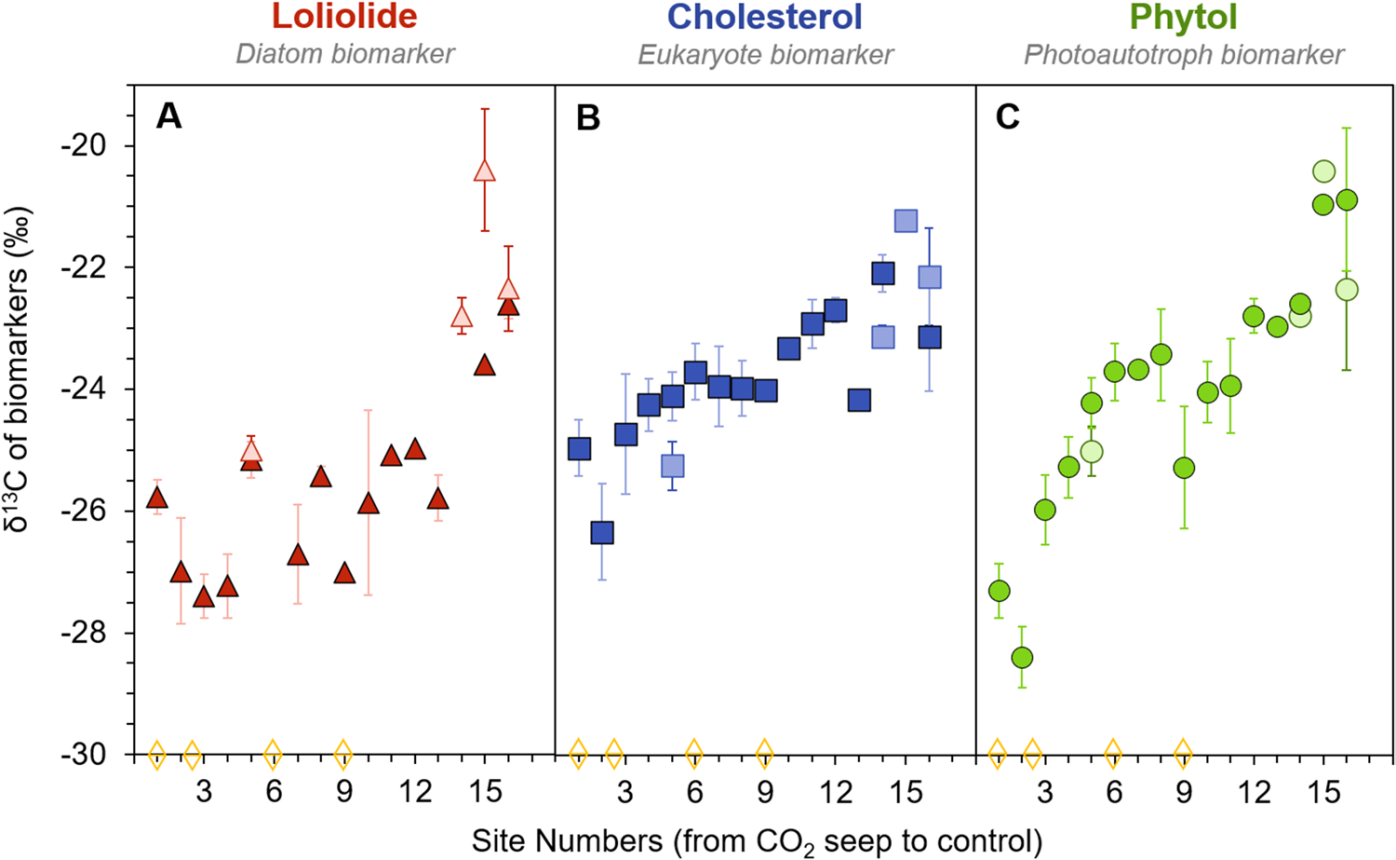
The δ^13^C of general phytoplankton biomarkers in surface sediments from Levante Bay, Italy. Loliolide (red triangle), cholesterol (blue square), and phytol (green circle) from CO_2_ seep (S) to control site (Site 16) sediment collected in May (light colors) and October (darker colors). Diamonds mark sites where there was minor additional bubbling of gas observed.

All three biomarkers show a steady increase in δ^13^C values over the transect from the CO_2_ seep towards the control site (Fig. 3; Table S2). The exceptions are the more depleted δ^13^C values at Site 2 and Site 9, where we observed some minor gas bubbling in the sediment, suggesting the release of small amounts of CO_2_ at these sites. Over the transect from Site 1 (the seep) to Site 16 (the control), the δ^13^C of loliolide ranges from − 27.4 to − 21.6‰ (Fig. 3A). From the seep to around Site 10, the δ^13^C of loliolide fluctuates between ca. − 27 and − 25‰, followed by a prominent increase from ca. − 25 to − 22.5‰. For the sites sampled in both May and October, there appears to be consistency between the two seasons, i.e. in Site 5 (− 25.2‰ for both seasons) and the control site (− 22.3‰ in May and − 22.6‰ in October), though Site 14 shows a spread of 2‰ between seasons. The δ^13^C of cholesterol shows a smaller but more consistent shift over the transect, ranging from − 26.3 to − 21.2‰ with a 1‰ difference between the two seasons (Fig. 3B). Phytol shows the largest shift, ranging from − 28.4‰ at the seep site to − 20.4‰ at the control site (Fig. 3C). There is a relatively consistent increase in the δ^13^C of phytol over the entire transect, except for a small decrease at Site 9, where we observed minor additional gas bubbling in the sediment. The δ^13^C of phytol shows minor variation between seasons (ca. 0.5%), except for the control site which showed a difference of 1.4‰.

## Discussion

The three most abundant biomarkers, loliolide, cholesterol, and phytol, are all derived from phytoplankton and represent broad phytoplankton groups^51–53^. Composition of the diatom assemblages and cyanobacteria in this bay are further described in Johnson et al.^43^ All become increasingly enriched in ^13^C over the transect from high CO_2_ concentrations near the seep to the control Mediterranean values. The observed isotopic depletion that occurs with increasing CO_2_ concentrations matches theory^5,6,54^. Furthermore, this pattern closely follows the results observed at Shikine Island, i.e. a consistent depletion δ^13^C of the same biomarkers with increasing proximity to the CO_2_ seep^50^, but here offered in a 16-point transect instead of the 3-points at the Japan site. Given that CO_2_ was the major variable over the transect in Italy, as well as Shikine Island, this strongly suggests that CO_2_ concentrations indeed have a strong impact on isotopic fractionation of general phytoplankton biomarkers, suggesting their potential as a *p*CO_2_ proxy.

Although the general trends between the two CO_2_ seep sites are similar, there is a difference in the magnitude and consistency in isotopic changes between the two sites. In the Shikine Island study, loliolide showed the largest isotopic shift over the transect (− 7.9‰) as compared with phytol (− 5.2‰) and cholesterol (− 5.2‰). However, in the Vulcano Island surface sediments, phytol had the most pronounced isotopic shift (− 8.0‰) as compared with loliolide (− 5.8‰) and cholesterol (− 5.1‰). Furthermore, the changes in loliolide over the Vulcano Island transect are more variable compared with the consistent trends in isotopic values observed in phytol and cholesterol. Here, we will explore these differences.

The δ^13^C profile of loliolide at Vulcano Island (Fig. 3A) has the least consistent trend among the three biomarkers, fluctuating between − 27.4 and − 25.0‰ from Site 1 (the seep) to Site 13. Loliolide is derived from the major xanthophyll fucoxanthin and is considered a biomarker for diatoms, especially in the absence of haptophyte algae^51,55^, based on its predominance at sites with substantial diatom communities, although some other non-diatom species also produce fucoxanthin^56^. Light microscopy analysis of selected sediments across the transect showed that Site 2 contains nearly no diatom frustules, Site 5 had abundant centric diatoms as well as some pennate diatoms, while Site 9 is characterized by a great diversity especially among pennate diatoms though with relatively low overall abundance, and Site 13 and Site 16 (control site) had both high abundance and high diversity of both centric and pennate diatoms (Stoll H. and Mejía Ramírez L. M., personal communications). Decreased diversity in increased proximity to the seep has previously been observed in periphytic diatom assemblages at this site^43^, though with a drastic increase abundance in chlorophyll-a by ca. fivefold from Site 6 to 16. Johnson et al. suggest that the increase abundance but decreased diversity is due to some diatoms benefitting from increasing CO_2_ through a reduction in the energetic costs of their CCMs^43^. The different composition of diatoms at each site, particularly between centric and pennate diatoms, may explain why we observe a high δ^13^C variability in loliolide. Different species may have slightly different isotopic fractionation due to e.g. different cell geometry and morphologies^11^ or different bicarbonate pumping strategies that has been observed in diatom species^57–59^. This concept may be further supported by the stronger increase in δ^13^C values observed between sites 13 and 16, where the higher diversity of species may yield a more robust overall δ^13^C signal through averaging biosynthetic differences among species. This complexity in the signal of loliolide may weaken the potential of this biomarker for past *p*CO_2_ reconstructions.

The δ^13^C profile of cholesterol (Fig. 3B) showed a more consistent decline over the transect than loliolide, though with a smaller difference in absolute values than phytol and loliolide from the seep towards the control. Because cholesterol is produced by all eukaryotes, such as phytoplankton or by heterotrophs, which modify ingested sterols^52,60^, terrestrial input, in addition to the algal input, can potentially dilute the autochthonous isotopic signal. However, the lack of terrestrial triterpenoids and long-chain (> C_22_) even carbon number fatty alcohols (Fig. 2) suggest minimal input of terrestrial biomarkers in the bay. Another explanation for the smaller isotopic change is that the cholesterol has contributions from heterotrophs, which produce cholesterol by modifying ingested phytoplanktonic sterols. Although this does not yield large isotopic fractionation^61^, the zooplankton often have stronger mobility than their photoautotroph counterparts; they may consume phytoplankton from various locations (and consequently various CO_2[aq]_ concentrations) throughout the bay. This idea is supported by the notable δ^13^C differences in cholesterol between the two seasons, where the offsets are not consistently in one direction. Based on these observations the δ^13^C of cholesterol must be considered carefully when used in reconstructing past CO_2_ concentrations.

The δ^13^C profile of phytol had the most robust trend across the transect (Fig. 3C) with an δ^13^C enrichment of ca. 8‰ from the seep to the control. Phytol, derived from chlorophyll-a, is found in all oxygenic photoautotrophs^53^. Terrestrial input may affect the signal of phytol but, as discussed above, there is no evidence for this here. Based on these results, phytol shows the greatest sensitivity to the CO_2_ gradient, and thus the most promise for reconstructing past *p*CO_2_. The phytol results from Shikine, Japan^50^ likewise show great promise for reconstructing past *p*CO_2_.

To test the validity of using the δ^13^C of the general biomarkers to estimate past *p*CO_2_, we used phytol, the most promising of the various general phytoplankton biomarkers explored here with the most consistent trend and the strongest δ^13^C shift over the gradient. We calculated the stable carbon isotopic photosynthetic fractionation (Ɛ_p_) using the δ^13^C of phytoplankton biomass (δ_p_) and the δ^13^C of CO_2_ (δ_d_):

2 $$ \upepsilon_{{\text{p}}} = 1000 \, \cdot \, \left[ { \, \left( {\delta_{{\text{d}}} + 1000} \right) \, / \, \left( {\delta_{{\text{p}}} + 1000} \right) \, {-}{ 1}} \right] $$

The δ_p_ is calculated from the offset between phytol and biomass, which is 3.5‰ ± 1.3 standard deviation based on the average of 23 representative marine phytoplankton species grown in cultures^39^. The δ_d_ is calculated from the δ^13^C of DIC (0.7‰ ± 0.4‰ s.d.) correcting for temperature and pH (Table S1). The mean annual sea surface temperature for Vulcano Island (20.2 ºC ± 0.5 °C s.d.; https://www.seatemperature.info) was used to calculate the temperature-dependent carbon isotopic fractionation of CO_2[aq]_ with respect to HCO_3_^–^^62^_._ The pH gradient, ranging from 5.5 pH near the vent to 8.2 pH in the control^63^, was used to define the relative contribution of different inorganic carbon species to the measured DIC^64^ (Table S1). Uncertainty was calculated using Monte Carlo simulations which consider the culmination of each individual parameter with its associated uncertainty, as described by Witkowski et al.^39^, here including δ^13^C of phytol ± 0.5‰ s.d., offset between biomass and phytol ± 1.3‰ s.d., δ_d_ ± 0.4‰ s.d., and T °C ± 0.5 °C (Table S2). This uncertainty has an equal effect on the final uncertainties in calculated ε_p_, i.e. 0.1‰ error in the δ_d_ will lead to a 0.1‰ error in ε_p_^39^.

**Figure 4:**
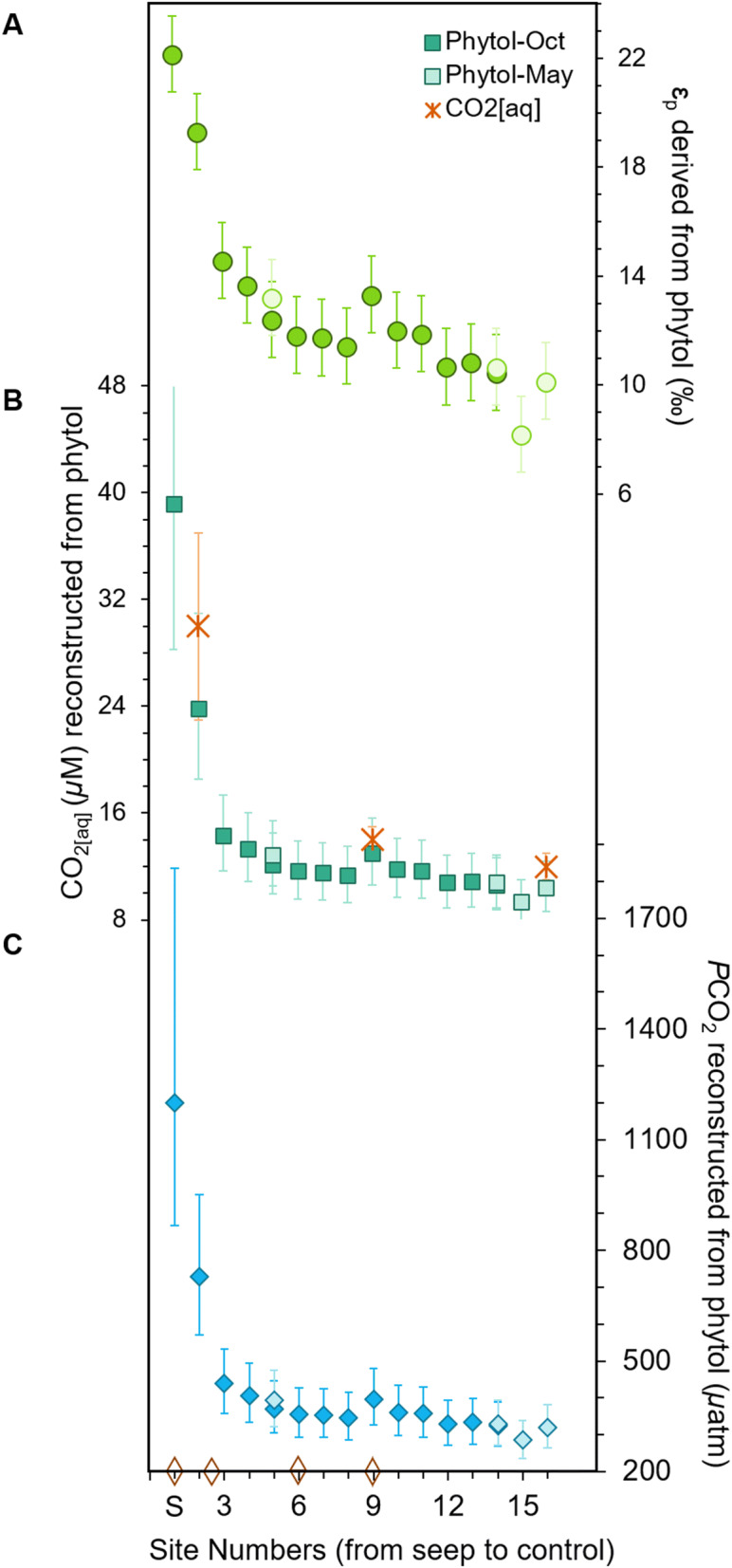
Ɛ_p_, CO_2[aq]_, and reconstructed *p*CO_2_ from phytol in surface sediments. (**A**) Ɛ_p_ of phytol, (**B**) CO_2[aq]_ based on Ɛ_p_ of phytol, and (**C**) *p*CO_2_ based on the δ^13^C of phytol from surface sediments collected in May (light colors) and October (dark colors), ranging from Site 1 (S) to Site 16 (control). Stars indicated CO_2_ concentrations^43^. Open orange diamonds mark regions where there was minor additional bubbling of gas.

Phytol-derived Ɛ_p_ ranges from 22.2 to 8.2‰ ± 1.4‰ s.d. (Fig. 4A) and shows a consistent decline in fractionation from the seep towards the control site. This includes Site 2 where measured δ^13^C values are higher than at Site 1, but Ɛ_p_ now shows the expected trend of more fractionation closer to the vent. This is attributed to the strong shift in pH between these two sites (5.5 pH at the vent and 6.25 pH at Site 2^63^) which we have here corrected for. The highest Ɛ_p_ value of 22.2‰ near the seep is approaching maximum isotopic fractionation due to CO_2_-fixation (Ɛ_f_), which has been shown to range between 25 and 28‰ in laboratory cultures^18^, but still does not quite reach full expression of Ɛ_f_. This is somewhat unexpected given the constant bubbling of CO_2_ at this site and thus very high CO_2_ concentrations, i.e. up to ca. 3× modern CO_2[aq]_^43^.

Several possibilities may explain why the full expression of Ɛ_f_ has not been reached. For one, given the relatively small area of the bay, it is possible that surface sediment has moved around the bay over time due to tidal actions and bottom water currents, which dampens the overall signal by allochthonous organic matter transported from area’s outside of the bay, as also inferred for Shikine Island^50^. Furthermore, algae are unlikely to grow and deposit in precisely same location and given that the impact of the CO_2_ seep noticeably changes over tens of meters^43^, this likely leads to some mixed signal among sites, resulting in a suppressed signal. Another alternative is that the calculated Ɛ_f_ of the phytoplankton community in Levante Bay may be lower than that inferred from the many culture studies^11,17,65^. Indeed, several recent studies show that Ɛ_f_ of the different Rubisco types may be lower than previously assumed^66^.

In order to see how well Ɛ_p_ of phytol can reconstruct CO_2[aq]_, we estimated CO_2[aq]_ and *p*CO_2_ from the δ^13^C of phytol using the equation adapted from the high plant model^5^ for algae^7^, and described in Eq. (1)^17^, where *b* reflects species carbon demand per supply^8^ and Ɛ_f_ reflects the maximum isotopic fractionation due to CO_2_-fixation. The value of *b* is a complicated catchall for factors influencing isotopic fractionation such growth rate and cell-size^67^, light intensity and membrane leakiness^24,68^, further complicated due to the multitude of sources for general phytoplankton biomarkers. Studies have suggested an empirical average 170‰ kg µM^−1^ ± 43 kg µM^−1^ s.d. for *b* based on a compilation of δ^13^C values of bulk organic matter in marine surface sediments, as well as some limited phytol studies^39,50^. Furthermore, we use an average Ɛ_f_ for phytoplankton species of 26.5‰ ± 1.5‰ uniform distribution^39^ based on the 25 to 28‰ range observed in laboratory cultures^69^. As described above, uncertainty was calculated using Monte Carlo simulations, considering each individual parameter with its associated uncertainty, as described by Witkowski et al.^39^. Here, we include the uncertainties associated with Ɛ_p_ plus the new additional uncertainties associated with *b* ± 43 kg µM^−1^ s.d., Ɛ_f_ ± 1.5‰ uniform distribution, T °C ± 0.5 °C s.d., and sea surface salinity ± 1‰ s.d.

The resulting phytol-based CO_2[aq]_ values range from 9.3 to 39.4 µM (Fig. 4B). The highest value of 39.2 µM (+ 20.6/− 11.0 µM) is near the vent at Site 1, dropping to 23.7 µM (+ 7.1/− 5.2 µM) at Site 2, then to 14.3 µM (+ 3.0/− 2.7 µM) at Site 3, before gently declining to 9.6 µM (± 1.8 µM) at the control Site 16. If we calculate the *p*CO_2_ from CO_2[aq]_ using Henry’s Law constant K_0_, which considers salinity and temperature^70^, the resulting *p*CO_2_ reconstruction range from 280 to 1,182 µatm (Fig. 4C). The highest *p*CO_2_ values were reconstructed for the sites closest to the seep, Site 1 at 1,200 µatm (+ 636/− 333 µatm) and Site 2 at 728 µatm (+ 222/− 158 µatm), while the remainder of the transect showed fairly ambient values from Site 3 at 438 µatm (+ 95/− 80 µatm) to the Site 16 control at 294 (+ 56/− 50 µatm).

Comparison of CO_2[aq]_ estimates with those reported for sites^63^ equivalent of our Site 2, 9, and 16 (30 µM ± 7, 14 µM ± 1, and 12 µM ± 1, respectively; Fig. 4B), show that these estimates agree within uncertainty, suggesting that our approach yields reasonable estimates. Only at the control site there is a slight underestimation of CO_2_ concentrations. One possible explanation is an incorrect assumption for the *b* value. However, this seems unlikely given that (i) *b* values would need to be increased beyond any known *b* value thus observed to account for this underestimation, and (ii) this would lead to even higher past *p*CO_2_ estimations which are based on *b* values compiled from laboratory cultures and natural experiments^39^. A more likely explanation is the change in phytoplankton community over the bay, where the control community is dominated by high affinity CCM species as observed for macroalgae^49^. Given that these species actively pump bicarbonate under low CO_2_ conditions, this may explain the lessened Ɛ_p_, yielding lower CO_2_ estimations. This effect has also been observed in the mesocosm experiments with different CO_2_ concentrations^40^, especially if there is limited carbon dioxide leakage from cells. Recent studies have shown lower sensitivity of Ɛ_p_ to CO_2_ in laboratory cultures and in glacial-interglacial reconstructions caused by the upregulation of phytoplankton CCMs^24,33^, which suggest using this Ɛ_p_ based proxy with caution in reconstructing low-CO_2_ worlds. In contrast, the proxy seems to do well in estimating *p*CO_2_ concentrations similar to some of the higher concentrations that have been reconstructed over the past 455 Myr^39^, suggesting it may be applicable for past greenhouse worlds.

## Conclusion

We tested three general phytoplankton biomarkers in surface sediments in a transect from a naturally occurring CO_2_ seep located in Levante bay, Vulcano Island, Italy, towards the open Tyrrhenian Sea. The δ^13^C of the biomarkers showed a distinct increase with increasing distance from the CO_2_ seep, in agreement with the idea that CO_2_ concentrations have a strong control on isotopic fractionation. In particular, the δ^13^C of phytol yielded a strong and consistent trend throughout the transect, and the agreement between estimated and measured CO_2_ concentrations demonstrates the promise of this biomarker for paleo *p*CO_2_ reconstructions. Our results show that CO_2_ seep environments may prove a useful testing ground for new CO_2_ proxies.

## Materials and methods

### Sample site

Levante Bay (Fig. 1) is located on the northeast of Vulcano Island, an Aeolian Island north of Sicily. Volcanic activity on the island started in the upper Pliocene^71^, where the cooling of magmatic and hydrothermal fluid mixing into the crater fumeroles is believed to have created the pocket of CO_2_, which outgasses into the bay^72^. Located at ca. 1 m depth at 38.41694° N 14.96° E, the main underwater venting gas field outputs ca. 3.6 tons of gas per day^73^. This gas is composed of 97–98% CO_2_ and ca. 2% H_2_S^63^. The sea water temperature^63^ of ca. 19.7 °C and salinity^43^ of ca. 38‰ is homogenous throughout the small bay. Currents are mostly wind-driven, with minimal tidal range (< 40 cm) and depths throughout the entire bay, and thus all sample sites, ranged between 1 and 2 m. Precipitation varies throughout the year, with the dry months (May–August) averaging 16 mm/month and the wet months (October–January) averaging 87 mm/month. The input of CO_2_ gas intensely influences the geochemical composition of the bay’s waters, as seen by the strong pH gradient starting at the seep to across the bay from pH 5.5 to 8.2 in April and from pH 6 to 8 in September. For more details on the geochemistry, see Boatta et al.^63^.

## Materials

Samples were collected in 23–24 May and 16–17 October of 2017. A preliminary study was conducted in May using one site with a high CO_2_ concentration, two sites with a middle CO_2_ concentration, and one control site (i.e. not affected by the CO_2_ seep) as defined in Johnson et al.^43^, where seawater was collected for the δ^13^C of dissolved inorganic carbon (DIC) and surface sediments were collected for the δ^13^C of biomarker lipids. Seawater for DIC analysis was collected by overfilling glass vials and adding mercury chloride (0.5%) before sealing the vials closed with Apiezon M grease and securing the stopper with rubber bands. Surface sediments were collected by diving, scooped into geochemical bags, and immediately frozen; once back in the lab, these sediments were freeze-dried and kept refrigerated. All surface sediments were collected in triplicate at each site within a square of 2 by 2 m. The same sediment sampling method was used again in October, when a higher-resolution transect of sixteen sites was collected (Fig. 1). Given that the results of the δ^13^C of DIC collected in May was homogenous throughout the bay (see Table S1), as also revealed by another study in this region^48^, seawater samples were not collected in October.

### Methods

The δ^13^C of DIC of seawater collected in May was measured on a gas bench coupled to an isotope ratio mass spectrometer (IRMS) in duplicate. Samples were prepared using 100 µL of 85% H_3_PO_4_ then flushed with He. Seawater (500 µL) was injected to each vial, left to react for 1 h, and then the headspace was measured. Standards prepared with 0.3 mg of Na_2_CO_3_ and 0.4 mg of Ca_2_CO_3_ were flushed with He, injected with 100 µL of 85% H_3_PO_4_, and reacted for 1 h. The standards were run at the start and end of each sequence, as well as every six runs.

Sediments were freeze-dried and homogenized using a mortar and pestle. Sediments were then extracted using a Dionex 250 accelerated solvent extractor at 7.6 × 106 Pa at 100 °C using dichloromethane (DCM): MeOH (9:1 v/v). Extracts were transferred to centrifuge tubes to be refluxed with 1 N KOH in MeOH and the resulting base hydrolyzed extracts were neutralized to pH 5 using 2 N HCl in MeOH. The hydrolyzed extract was separated into apolar (hexane: DCM, 9:1 v/v), ketone (DCM), and polar (DCM: MeOH, 1:1 v/v) fractions, respectively, over an alumina column. Polar fractions were silylated with pyridine: N,O-Bis(trimethylsilyl) trifluoroacetamide (1:1 v/v) and heated for 1 h at 60 °C. The δ^13^C values of loliolide, cholesterol, and phytol were corrected for the addition of three C atoms in the trimethylsilyl group using the known δ^13^C value of BSTFA (− 32.2‰).

Silylated polar fractions were then injected on gas chromatography-flame ionization detector (GC-FID) to determine relative abundances and general quality of chromatography before analyzing it on a gas chromatography–mass spectrometer (GC–MS) to identify compounds and on gas chromatography-isotope ratio-mass spectrometer (GC-IRMS) to measure the isotopic composition of specific compounds. GC-FID, GC–MS, and IRMS instrumentation all had starting oven temperatures of 70 ºC ramped at 20 ºC/min to 130 ºC and then ramped at 4 ºC/min to 320 ºC for 10 min. Separation was accomplished using a CP-Sil 5 column (25 m × 0.32 mm; df 0.12 μm) with He carrier gas. System performance on all three instruments was conducted daily using the same in-house mixture of *n-*alkanes and fatty acids. Additional standards were run on the IRMS using perdeuterated *n*-alkanes (C_20_ and C_24_) with known δ^13^C values (− 32.7 and − 27.0‰, respectively) and were limited to uncertainty within the standard of ± 0.5‰; if outside this range, the machine was conditioned until it was within this limit. The IRMS was also oxidized regularly, with a daily oxidation of 10 min, backflushed with He for 10 min, and purged for 5 min; a shorter version of this sequence was conducted in post-sample seed oxidation, which includes 2 min oxidation, 2 min He backflush, and 2 min purge conditioning line and a longer version of this sequence was conducted at the end of each week with 1 h oxidation, 1 h He backflush, and 10 min purge conditioning line.

## Supplementary information

Supplementary Tables.

## Data Availability

All data are present in the paper and/or the Supplementary Materials.
